# Isotherm, Kinetics and Thermodynamics of Cu(II) and Pb(II) Adsorption on Groundwater Treatment Sludge-Derived Manganese Dioxide for Wastewater Treatment Applications

**DOI:** 10.3390/ijerph18063050

**Published:** 2021-03-16

**Authors:** Stephanie B. Tumampos, Benny Marie B. Ensano, Sheila Mae B. Pingul-Ong, Dennis C. Ong, Chi-Chuan Kan, Jurng-Jae Yee, Mark Daniel G. de Luna

**Affiliations:** 1 Environmental Engineering Program, National Graduate School of Engineering, University of the Philippines Diliman, Quezon City 1101, Philippines; tumamposstephanie@gmail.com (S.B.T.); sheila.pingul.ong@gmail.com (S.M.B.P.-O.); dcong@up.edu.ph (D.C.O.); 2 University Core Research Center for Disaster-free and Safe Ocean City Construction, Dong-A University, Busan 49315, Korea; bmensano@dau.ac.kr; 3 School of Technology, University of the Philippines Visayas, Miagao, Iloilo 5023, Philippines; 4 Institute of Hot Spring Industry, Chia-Nan University of Pharmacy and Science, Tainan 71710, Taiwan; cckanev@mail.cnu.edu.tw; 5 Department of Architectural Engineering, Dong-A University, Busan 49315, Korea; 6 Department of Chemical Engineering, University of the Philippines Diliman, Quezon City 1101, Philippines

**Keywords:** groundwater treatment sludge, heavy metal adsorption, isotherm, kinetics, manganese dioxide, thermodynamics

## Abstract

The ubiquitous occurrence of heavy metals in the aquatic environment remains a serious environmental and health issue. The recovery of metals from wastes and their use for the abatement of toxic heavy metals from contaminated waters appear to be practical approaches. In this study, manganese was recovered from groundwater treatment sludge via reductive acid leaching and converted into spherical aggregates of high-purity MnO_2_. The as-synthesized MnO_2_ was used to adsorb Cu(II) and Pb(II) from single-component metal solutions. High metal uptake of 119.90 mg g^−1^ for Cu(II) and 177.89 mg g^−1^ for Pb(II) was attained at initial metal ion concentration, solution pH, and temperature of 200 mg L^−1^, 5.0, and 25 °C, respectively. The Langmuir isotherm model best described the equilibrium metal adsorption, indicating that a single layer of Cu(II) or Pb(II) was formed on the surface of the MnO_2_ adsorbent. The pseudo-second-order model adequately fit the Cu(II) and Pb(II) kinetic data confirming that chemisorption was the rate-limiting step. Thermodynamic studies revealed that Cu(II) or Pb(II) adsorption onto MnO_2_ was spontaneous, endothermic, and had increased randomness. Overall, the use of MnO_2_ prepared from groundwater treatment sludge is an effective, economical, and environmentally sustainable substitute to expensive reagents for toxic metal ion removal from water matrices.

## 1. Introduction

Heavy metals are among the recalcitrant pollutants currently affecting the global human population [1]. These pollutants are produced in large quantities by anthropogenic activities and are inadequately removed by conventional water treatment processes [2,3]. When released to recipient waters and ingested by aquatic and terrestrial organisms, these pollutants may give rise to serious human and animal health problems. In trace amounts, some heavy metals such as manganese and copper are considered essential nutrients owing to their vital roles in several physiological processes [4]. However, chronic exposure to these metals may result in progressive neurodegenerative disorders such as manganism, Parkinson’s disease, and Alzheimer’s disease [5,6]. Other heavy metal ions such as Pb(II) and As(V) do not have biological functions and are extremely toxic even at minuscule amounts [7,8]. For example, acute and chronic Pb(II) exposure can have detrimental effects on the skeletal, immune, cardiovascular, renal, neurological, endocrine, respiratory, gastrointestinal, and reproductive systems [9]. Thus, removal of these toxic metals from drinking water sources is needed to ensure public health and safety.

Manganese is a naturally occurring transition metal found in groundwater sources [10,11]. During drinking water production, manganese is extracted from groundwater through a series of physicochemical processes (i.e., aeration/oxidation, sand filtration, and backwashing), producing large quantities of Mn-laden sludge, which are either disposed in landfills or dumped into water reservoirs. Current sludge management practices are not environmentally sustainable since manganese can still be further recovered from groundwater treatment sludge for future recycling applications.

Reductive acid leaching was employed to recover manganese from iron ores [12]. Due to its environmental compatibility and high efficiency in recovering metals while requiring low chemical consumption, reductive acid leaching was subsequently applied to extract valuable metals from secondary sources [13]. The leaching process uses strong acids and reducing agents for enhanced recovery of metal ions from solid samples. Recently, reductive acid leaching, using sulfuric acid as the leaching agent and hydrogen peroxide as the reductant, was used to extract manganese from groundwater treatment sludge, wherein 100% *Mn* recovery was achieved [14]. During reductive acid leaching, manganese in the sludge in the form of *MnO*_2_ is reduced to *Mn*^2+^ according to Equation (1):

(1) $$MnO_{2}+H_{2}O_{2}+2H^{+}\to Mn^{2+}+2H_{2}O+O_{2}$$

A previous study reported that the reaction between *MnO*_2_ and *H*_2_*SO*_4_ is not spontaneous and that *MnO*_2_ is insoluble in *H*_2_*SO*_4_ solution [12]. However, the addition of *H*_2_*O*_2_ enhances the dissolution of *MnO*_2_ in *H*_2_*SO*_4_ solution and improves the leaching efficiency. Other manganese species present in the sludge such as *MnO* can completely react with sulfuric acid spontaneously (Equation (2)), but *Mn*_2_*O*_3_ and *Mn*_3_*O*_4_ only partially react with the acid (Equations (3) and (4)) to produce *MnO*_2_, which can be further reduced to *Mn*^2+^ upon *H*_2_*O*_2_ addition.

(2) $$MnO+H_{2}SO_{4}\to MnSO_{4}+H_{2}O$$

(3) $$Mn_{2}O_{3}+H_{2}SO_{4}\to MnO_{2}+MnSO_{4}+H_{2}O$$

(4) $$Mn_{3}O_{4}+2H_{2}SO_{4}\to MnO_{2}+2MnSO_{4}+2H_{2}O$$

The recovery of heavy metals from wastes and the use of recovered metals in removing other heavy metals from water matrices are sustainable and environmentally-sound strategies. One simple but highly promising use of recovered manganese is its conversion to highly-efficient adsorbents for environmental contaminants. Manganese can be converted to *MnO*_2_ adsorbent by oxidation with potassium permanganate. Compared with commercially-available reagent-grade *MnO*_2_, sludge-derived *MnO*_2_ is highly economical. The estimated production cost of *MnO*_2_ by reductive leaching, hydroxide precipitation, and permanganate reduction is only USD 151.67 kg^−1^, less costly than the USD 222.08 kg^−1^ reagent-grade *MnO*_2_ [15]. This demonstrates the potential for sludge-derived *MnO*_2_ to be used as an inexpensive adsorbent for heavy metal abatement in contaminated waters.

In this study, *MnO*_2_ was synthesized from groundwater treatment sludge and its potential for wastewater treatment application was examined. The use of sludge-derived *MnO*_2_ for the removal of Cu(II) and Pb(II) from single-component metal solutions has scarcely been reported in literature. Hence, the main goal of the study is to present a quantitative and mechanistic description of Cu(II) and Pb(II) uptake from single-component metal solutions using the as-synthesized *MnO*_2_ adsorbent. Specifically, metal uptake was evaluated at varying initial pH, initial metal concentrations, and solution temperatures to estimate the relative influence of these parameters on the adsorption process. Langmuir, Freundlich, Temkin, and Dubinin–Radushkevich models were fitted to the equilibrium adsorption data to analyze the sorbate—sorbent interactions. Pseudo-first-order and pseudo-second-order kinetic models were also applied to the experimental data to determine the rate-limiting step in the adsorption process. Lastly, the thermodynamics of metal uptake by *MnO*_2_ was investigated to assess the spontaneity of the process. Information obtained in this study can be useful for future upscale studies featuring sludge-derived *MnO*_2_ as a heavy metal adsorbent. The initial investigations on single-component solutions can aid in assessing if the adsorptive performance of the sludge-derived *MnO*_2_ is significantly modified due to the competition and interaction among the individual metal species, especially when applied to industrial wastewater containing a mixture of toxic heavy metals.

## 2. Materials and Methods

### 2.1. Chemicals and Materials

Analytical grade chemicals were used as received and without further purification. Copper(II) sulfate (CuSO_4_, 99%, Merck, Darmstadt, Germany) and lead(II) nitrate (Pb(NO_3_)_2_, 99%, Merck, Darmstadt, Germany) were used in the preparation of metal solutions. Manganese from groundwater treatment sludge was extracted and converted to MnO_2_ using sulfuric acid (H_2_SO_4_, 95–97%, Merck, Darmstadt, Germany), hydrogen peroxide (H_2_O_2_, 35%, Shimakyu, Osaka, Japan), sodium hydroxide (NaOH, >98%, Shimakyu, Osaka, Japan), and potassium permanganate (KMnO_4_, 99%, J.T. Baker, Phillipsburg, NJ, USA). All solutions were prepared with deionized water (>18.2 MΩ cm resistivity) from a Purelab deionizer (ELGA LabWater, High Wycombe, UK).

Groundwater treatment sludge was sourced from Changhua Waterworks Third Water Purification Plant in Changhua County, Taiwan, and dried for 24 h at 105 °C in a laboratory oven (UFE 400, Memmert GmbH + Co. KG, Schwabach, Germany). Thereafter, the sludge was crushed with a mortar and pestle, sieved using mesh #50 with 0.297 mm opening, and stored for Mn extraction.

### 2.2. Mn Leaching and MnO_2_ Synthesis

The sludge-derived MnO_2_ was synthesized following the method reported in previous studies [14,15,16]. Predetermined volumes of 0.8 M H_2_SO_4_ and 0.8 M H_2_O_2_ were added to the dried sludge in a 250 mL Erlenmeyer flask, and the mixture was stirred on a magnetic stirrer and hot plate (PC-420D, Corning, NY, USA) for 5 min at 150 rpm and 25 °C. The mixture was filtered through 110 mm Whatman filter paper, after which small drops of 1 M NaOH were carefully added to the filtrate while it was stirred at 300 rpm and 25 °C. This was continued until the solution pH of 4.0 was reached ensuring complete precipitation of Fe^3+^ in the solution [14]. Next, the Fe^3+^ residue was separated from the solution via filtration using 110 mm Whatman filter paper leaving only Mn^2+^ in the filtrate. A 0.02 M KMnO_4_ solution was then slowly added to the filtrate under constant agitation of 300 rpm at 90 °C. The resulting purple mixture was filtered through 110 mm Whatman filter paper, and the precipitate (MnO_2_) was dried for 24 h at 105 °C.

### 2.3. Adsorption Experiments

Batch experiments were carried out to examine the adsorption of Cu(II) or Pb(II) on MnO_2_. First, a 30 mL single-component metal ion solution was prepared in a 125 mL Erlenmeyer flask via dilution of Cu(II) or Pb(II) stock solutions with deionized water. The solution pH was monitored using a pH meter (PC-310, Suntex Instruments Co., Ltd., New Taipei City, Taiwan) and adjusted by dropwise addition of 0.1 M NaOH and 0.1 M HCl. Then, a predetermined amount of MnO_2_ was added and the mixture was agitated in a water bath shaker (SB303, Kansin Instruments Co., Ltd., New Taipei City, Taiwan) at 50 rpm for 24 h. After each run, samples were taken using a 10 mL Terumo syringe and were filtered with microsyringe filters (0.45 μm, Minisart NY 25, Sartorius Stedim Biotech, Goettingen, Germany). The filtrates were then analyzed for metal ion concentration using an inductively coupled plasma mass spectrometer (ICP-MS, Optima 5300 DV, Perkin Elmer, Waltham, MA, USA). The adsorption capacity (*q_e_*, mg g^−1^) was calculated using Equation (5).

(5) $$q_{e}=\frac{\left(C_{o}-C_{e}\right)\left(V\right)}{\left(m\right)}$$

where *Co* is the initial concentration of the metal ion, *Ce* is the concentration of the metal ion in equilibrium (mg L^−1^); *V* is the volume of the solution in liters (L); *m* is the adsorbent dosage (g).

Experimental parameters such as initial metal concentration, solution pH, and temperature were varied to determine the maximum potential of the adsorbent. Initial conditions were set at 50 rpm agitation speed, 30 mL adsorbate volume, and 24 h contact time. Using the optimum pH and temperature obtained from the parametric study, isotherm studies were conducted at various initial metal ion concentrations (10, 50, 75, 100, 125, 150, and 200 mg L^−1^) and results were plotted using Langmuir, Freundlich, Temkin, and Dubinin–Radushkevich models. Kinetic studies were carried out at predetermined durations from 1 to 24 h using 100 mg L^−1^ adsorbate concentrations, and the experimental data were fitted to pseudo-first-order and pseudo-second-order kinetic models. The thermodynamics of Cu(II) or Pb(II) adsorption on the as-prepared MnO_2_ adsorbent was evaluated by predetermined solution temperature from 25 to 35 °C.

### 2.4. Adsorbent Characterization

A scanning electron microscope with energy-dispersive X-ray spectrometer (SEM-EDS, JSM-5310, JEOL, Tokyo, Japan) and a Fourier transform infrared spectrometer (FTIR, Nicolet 6700, Thermo Electron Corp., Madison, WI, USA) were used to analyze the surface morphology and functional groups of the as-prepared adsorbent, respectively. An N5 submicron particle size analyzer (Beckman Coulter, Miami, FL, USA) was used to determine the particle size distribution of the adsorbents.

## 3. Results and Discussion

### 3.1. Adsorbent Properties

**Figure 1:**
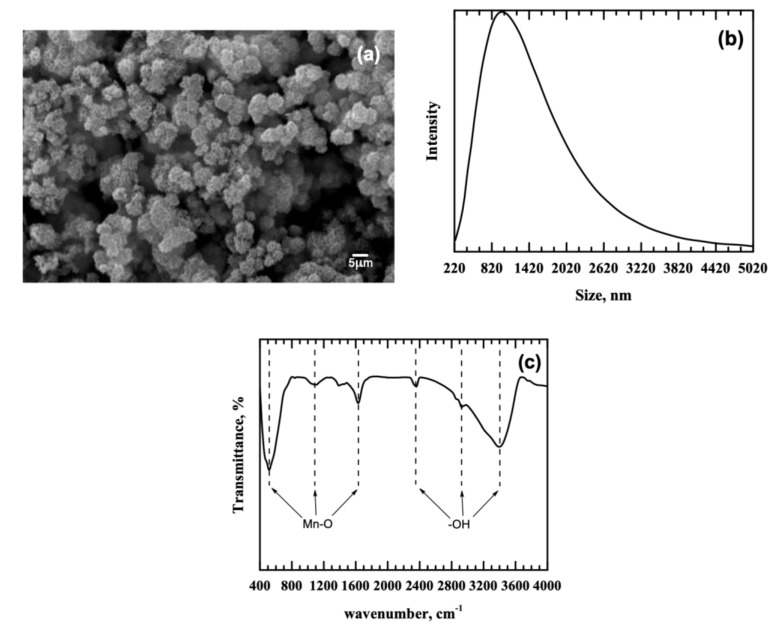
Characteristics of MnO_2_: (**a**) morphology, (**b**) particle size distribution, and (**c**) functional groups.

Figure 1a presents the SEM image of the MnO_2_ adsorbent derived from the groundwater treatment sludge. The surface morphology of MnO_2_ is characterized by spherical agglomerates, which can be attributed to the preparation conditions. Spherical agglomerates develop at a low-aging temperature (25 °C) and a short duration (4 h), whereas nanorod-like structures are formed at a higher temperature (80 °C) and longer duration (8 h) [17]. This feature is similar to the particle clusters noted in the raw sludge [11], ascribed to precipitated manganese and iron, but with larger sand particles removed through reductive acid leaching process [14]. EDS analysis of MnO_2_ revealed the presence of oxygen (27.53%) and manganese (72.47%), confirming the high-purity MnO_2_ produced and the successful Mn recovery from groundwater treatment sludge via reductive acid leaching. In Figure 1b, the majority of the MnO_2_ agglomerates was in the size range of 820–1420 nm, and the average particle size was 1105.3 nm. The FTIR spectrum of MnO_2_ is shown in Figure 1c. Prominent peaks are observed at 516, 1098, 1631, 2358, 2926, and 3396 cm^−1^, which correspond to Mn−O stretching vibrations, O−H bending vibrations attached to Mn atoms, and O−H stretching vibrations of adsorbed water [18]. Similar peaks were noted in the raw sludge where hydroxyl functional groups associated with Mn atoms and water molecules were present [11].

### 3.2. Effects of pH, Initial Metal Concentration, and Temperature

**Figure 2:**
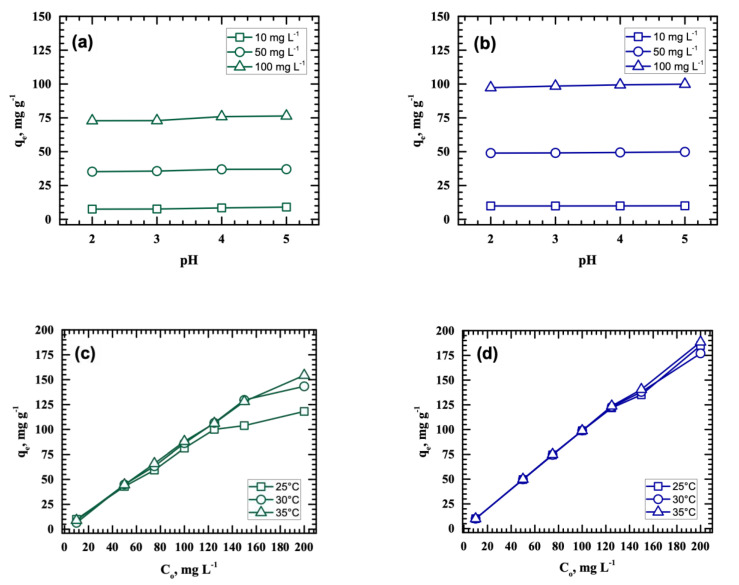
Effect of initial pH, metal concentration, and temperature on the uptake of Cu(II) (**a**,**c**) and Pb(II) (**b**,**d**) by MnO_2_.

The behavior of the Cu(II) and Pb(II) adsorption capacities of MnO_2_ at varying initial solution pH (pH 2 to 5) and initial metal ion concentration (10, 50, and 100 mg L^−1^ Cu(II) or Pb(II)) are depicted in Figure 2a,b. Agitation speed, solution temperature, and contact time were maintained at 50 rpm, 25 °C, and 24 h, respectively. The metal solutions were examined under pH 2 to 5 because precipitations of Cu(II) and Pb(II) species occur at pH > 5 [19,20]. In the figure, only slight increases in the amount of metal adsorbed per unit mass of MnO_2_ were noted as initial solution pH was raised from 2 to 5. Specifically, Cu(II) adsorption capacity increased by 6.28%, while Pb(II) uptake improved by 1.08% when the metal ion concentration was kept at 10 mg L^−1^. At 50 mg L^−1^, Cu(II) adsorption capacity slightly improved by 5.14%, while Pb(II) uptake increased by 1.76%. Lastly, when the metal ion concentration was 100 mg L^−1^, improvements in the adsorption capacities for Cu(II) and Pb(II) were noted at 4.82% and 2.63%, respectively. Clearly, Cu(II) is more sensitive to pH change than Pb(II). The results shown in Figure 2a,b are similar to a previous study [15], and the removal of Cu(II) and Pb(II) from the aqueous solutions can be attributed to the fixed amount of negative surface charge on the MnO_2_ that interacted with the metal ions at all pH levels. Although the sludge-derived adsorbent had a pH_PZC_ of 6.0 [16], the metal uptake results confirmed that MnO_2_ remained effective in adsorbing Cu(II) and Pb(II) even at lower pH levels.

Figure 2a,b also show that Cu(II) and Pb(II) uptake improved as the initial metal concentrations were increased at all pH levels. At 100 mg L^−1^, maximum Cu(II) (76.37 mg g^−1^) and Pb(II) (99.87 mg g^−1^) adsorption capacities were obtained, which were approximately 2 and 10× the adsorption capacities reached when the metal ion concentrations were 50 and 10 mg L^−1^, respectively. This indicates that increasing the amount of metal ions improved the interaction between the Cu(II) or Pb(II) and the surface functional groups of the adsorbent, which enhanced metal ion removals. Moreover, in all initial metal ion concentrations examined, Pb(II) adsorption was consistently higher than Cu(II). The ionic potentials of Cu(II) and Pb(II) are 2.30 and 1.50, respectively. As Cu(II) has a higher ionic potential, its capacity to repel H^+^ ions on MnO_2_ is higher than that of Pb(II). This gives Pb(II) a greater tendency to be adsorbed on the surface of MnO_2_ than Cu(II). Solution temperature also affects the process of metal ion adsorption. In Figure 2c,d, Pb(II) and Cu(II) adsorption capacities improved as the temperature was increased, indicating that the adsorption process is an endothermic reaction. The direct correlation of solution temperature with the amount of metals adsorbed can be attributed to the faster rate of ion diffusion at a higher temperature [21].

### 3.3. Isotherm Studies

The mechanism of interactions between the metal adsorbates and the surface functional groups of MnO_2_ can be better understood by analyzing Pb(II) and Cu(II) removal data using the Langmuir [22], Freundlich [23], Temkin [24], and Dubinin–Radushkevich [25] isotherm models expressed in Equations (6)–(9), respectively:

(6) $$q_{e}=\frac{q_{m}K_{L}C_{e}}{1+K_{L}C_{e}}$$

(7) $$q_{e}=K_{F}C_{e}{}^{1/n}$$

(8) $$q_{e}=Bln\left(K_{T}C_{e}\right)$$

(9) $$q_{e}=q_{DR}e^{-K_{DR}\varepsilon^{2}}$$

where *C_e_* is the equilibrium concentration of the adsorbate in mg L^−1^, *q_e_* is the amount of adsorbate adsorbed per gram of the adsorbent at equilibrium in mg g^−1^, *q_m_* is the maximum monolayer-coverage capacity in mg g^−1^, *K_L_* is the Langmuir isotherm constant, *K_f_* and *n* are Freundlich constants that encompass all parameters in the process such as adsorption capacity and intensity, *B* = (RT/$b_{t}$; in J mol^−1^) corresponds to the heat of adsorption, *T* is the absolute temperature, *R* is the ideal gas constant, $b_{t}$ is the Temkin constant, *A* is the equilibrium binding constant that corresponds to the maximum binding energy, *q_DR_* (mg g^−1^) is the adsorption capacity, *K_DR_* (mol^2^ kJ^−2^) is a constant related to the sorption energy, and *ε* is the Polanyi potential defined by Equation (10):

(10) $$\varepsilon =RT\ln\left[1+\frac{1}{C_{e}}\right]$$

**Figure 3:**
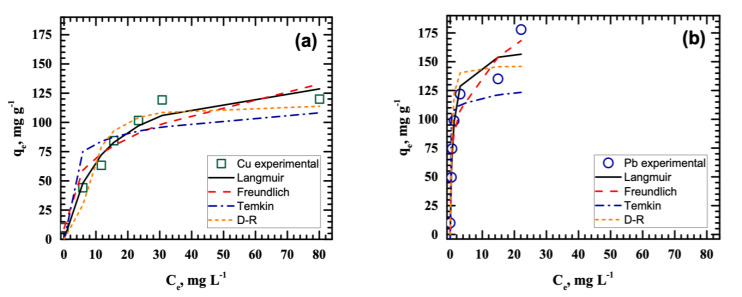
Langmuir, Freundlich, Temkin, and Dubinin–Radushkevich nonlinear isotherm plots for the removal of (**a**) Cu(II) and (**b**) Pb(II).

Experimental Cu(II) and Pb(II) adsorption data fitted to select isotherm models are presented in Figure 3, and the calculated isotherm parameters are listed in Table 1. Results show that Cu(II) and Pb(II) removal conformed to the Langmuir model since the values of the coefficients of determination (*R*^2^) were highest in Langmuir compared with the other models. This means that Cu(II) and Pb(II) uptake by MnO_2_ is dominated by monolayer adsorption, in agreement with the results of a previous study [15]. Herein, adsorbate removal was facilitated by the attraction between the positively-charged metal ions and the negatively-charged binding sites on the sludge-derived MnO_2_, resulting from the loss of H^+^ in hydrated MnO_2_ when suspended in water [26]. The active sites with uniform energy levels on the hydrated MnO_2,_ as confirmed by surface hydroxyl groups, bind with Cu(II) or Pb(II) in a single-layer arrangement via ion-exchange adsorption or complexation until the binding sites become saturated and no further adsorption can occur on the same site.

The adsorption process following the Langmuir model was further validated using the separation factor *R_L_*_,_ computed using Equation (11). *R_L_*_,_ is the equilibrium parameter that describes the nature of the adsorption process, and is expressed in a dimensionless constant [27]. Depending on the *R_L_* value, the adsorption process can be unfavorable (*R_L_* > 1), linear (*R_L_* = 1), favorable (0 < *R_L_* < 1), or irreversible (*R_L_* = 0) [28]. The results in Table 1 show that the *R_L_* values were below one but greater than zero for Cu(II) (0.0583–0.5530) and Pb(II) (0.0040–0.0746). These results confirm the suitability of as-synthesized MnO_2_ as adsorbent for the said metal ions.

(11) $$R_{L}=\frac{1}{1+C_{o}K_{L}}$$

### 3.4. Kinetic Studies

The pseudo-first-order [29] and the pseudo-second-order [30] kinetic models, expressed in Equations (12) and (13), respectively, were used to determine the rate-determining step of the adsorption process:

(12) $$q_{t}=q_{e}\left(1-e^{-k_{1}t}\right)$$

(13) $$q_{t}=\frac{q_{e}{}^{2}k_{2}t}{1+k_{2}q_{e}t}$$

where $k_{1}$ is the pseudo-first-order rate constant per minute, $k_{2}$ is the rate constant of pseudo-second-order adsorption (g mg^−1^ min^−1^), $q_{e}$ is the amount of adsorbate adsorbed at equilibrium per unit weight of the adsorbent (mg g^−1^), and $q_{t}$ is the amount of adsorbate adsorbed at any time (mg g^−1^).

**Figure 4:**
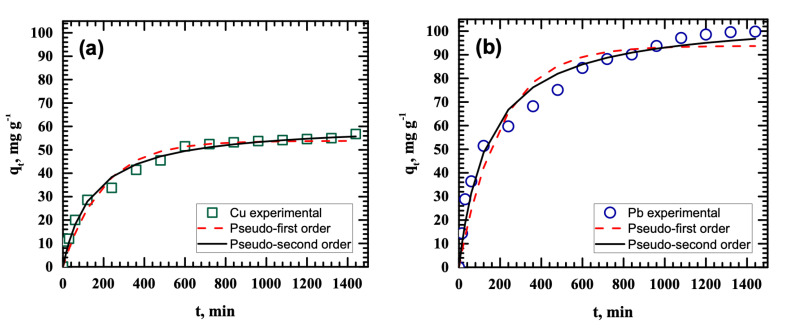
Pseudo-first- and pseudo-second-order nonlinear kinetic plots for the removal of (**a**) Cu(II) and (**b**) Pb(II).

Figure 4 presents the pseudo-first- and pseudo-second-order nonlinear kinetic plots for the removal of Cu(II) and Pb(II), and Table 2 summarizes the calculated kinetic parameters. Based on the *R*^2^ values, the pseudo-second-order kinetic model conformed to the experimental data better than the pseudo-first-order kinetic model. The values of the experimental adsorption capacities of Cu(II) and Pb(II) agree with the calculated pseudo-second-order parameters. These results indicate that the rate-limiting step for the adsorption of Pb(II) and Cu(II) on MnO_2_ is chemical adsorption via the formation of metal–MnO_2_ complexes at the functional groups on the adsorbent surface [15].

Table 3 presents a comparison of the findings of the present work in terms of maximum adsorption capacity with other materials employed for Cu(II) and Pb(II) removal. As shown, the q_max_ values of sludge-derived MnO_2_ are notably higher than those reported in other studies. This confirms that the sludge-derived MnO_2_ in the present study is a competitive adsorbent for Cu(II) and Pb(II).

### 3.5. Thermodynamic Studies

The spontaneity, thermal feasibility, and nature of reaction during Cu(II) and Pb(II) adsorption by MnO_2_ were examined using the standard Gibb’s free energy change (Δ*G°*), change in enthalpy (Δ*H°*), and change in entropy (Δ*S°*) following Equations (14)–(17):

(14) $$\Delta G^{{}^{\circ}}=\Delta H^{{}^{\circ}}-T\Delta S^{{}^{\circ}}$$

(15) $$\Delta G^{{}^{\circ}}=-RTlnK_{e}$$

(16) $$K_{e}=\frac{C_{a}}{C_{e}}$$

(17) $$lnK_{e}=\frac{\Delta S^{{}^{\circ}}}{R}-\frac{\Delta H^{{}^{\circ}}}{RT}$$

where *C_a_* is the amount of metal adsorbed at equilibrium (mg L^−1^), *C_e_* is the equilibrium metal concentration (mg L^−1^), *R* is the universal gas constant (J mol·K^−1^), and *T* is the absolute temperature (K). Δ*H°* and Δ*S°* are obtained from the slope and intercept of a plot of *ln K_e_* versus *1/T*.

The activation energy, *E_a_* (kJ mol^−1^), which determines the temperature dependence of the reaction rate, was computed from the Arrhenius equation in Equation (18), where *k*_2_ is the pseudo-second-order rate constant (g mg^−1^ min^−1^), and *k_o_* is a temperature-independent parameter (g mg^−1^ min^−1^).

(18) $$lnk_{2}=-\frac{E_{a}}{RT}+lnk_{0}$$

Table 4 presents the thermodynamic parameters of Cu(II) and Pb(II) adsorption by MnO_2_. The calculated negative *ΔG°* corresponds to the spontaneous and thermodynamically favorable Cu(II) and Pb(II) adsorption process at 25–35 °C [36]. The increasing magnitude of Δ*G°* with the rise in temperature indicates more favorable adsorption at higher temperatures. Positive Δ*H°* values confirm the endothermic nature of the adsorption process [37]. The Δ*S°* values for Cu(II) and Pb(II) adsorption were positive, which denoted an increase in randomness of the solid–solution interface and a strong affinity between the metal ions and MnO_2_ [38]. The energy of activation was calculated using the slope of the Arrhenius plot [39]. The activation energies of adsorption of Cu(II) and Pb(II) were computed at 146.76 and 130.13 kJ mol^−1^, respectively. These results confirm chemical adsorption, and high values of activation energy indicate that diffusion is not a rate-limiting factor in the adsorption process [40].

## 4. Conclusions

In this study, manganese oxide derived from groundwater treatment sludge was used for Cu(II) and Pb(II) removal from single metal solutions. SEM and particle size analyses of the MnO_2_ revealed the presence of spherical agglomerates of MnO_2_ particles with an average particle size of 1105.3 nm. FTIR results showed stretching and bending vibrations of Mn−O and Mn−OH functional groups at the adsorbent surface, which are responsible for the uptake of Cu(II) and Pb(II) from aqueous solutions. Langmuir and pseudo-second-order models conformed to experimental data, signifying a monolayer coverage and chemical adsorption process, respectively, and these findings were confirmed by the high magnitude of the activation energies for Cu(II) (146.76 kJ mol^−1^) and Pb(II) (130.13 kJ mol^−1^). Cu(II) and Pb(II) sorption onto MnO_2_ was found to be spontaneous, endothermic, and increasingly random. Overall, the study was able to demonstrate that groundwater treatment sludge can be an inexpensive raw material for the production of highly-efficient adsorbents for the removal of Cu(II) and Pb(II) from aqueous systems. The results expand the applicability of sludge-derived MnO_2_ and thus establish groundwater treatment sludge as a viable and environmentally-sound secondary source of manganese for various water treatment applications. Future upscale studies based on the laboratory results are recommended.

## Figures and Tables

**Table 1:** Isotherm parameters for metal uptake.

Isotherm Model	Parameter	Cu(II)	Pb(II)
Langmuir	*q_m_* exp(mg g^−1^)	119.90	177.89
	*q_m_* (mg g^−1^)	148.58	162.19
	*K_L_* (L mg^−1^)	0.0808	1.2396
	*R_L_*	0.0583–0.5530	0.0040–0.0746
	*R* ^2^	0.9534	0.9460
Freundlich	*n*	3.2331	4.4354
	*K_F_* (mg^1−1/n^ L^1/n^ g^−1^)	34.2414	83.7481
	*R* ^2^	0.8952	0.9381
Temkin	*B* (J mol^−1^)	12.70	5.3278
	*K_T_* (L g^−1^)	62.88	5.1539 × 10^8^
	*R* ^2^	0.7748	0.6245
Dubinin–Radushkevich	*K_DR_* (mol^2^ kJ^−2^)	9.01 × 10^−6^	8.04 × 10^−8^
	*q_DR_* (mg g^−1^)	114.77	145.99
	*R* ^2^	0.9207	0.8637

**Table 2:** Kinetic parameters for metal uptake.

Kinetic Model	Parameter	Cu(II)	Pb(II)
Pseudo-first-order	*q_e_* exp (mg·g^−1^)	56.74	99.80
	*q_e_* (mg·g^−1^)	53.82	93.74
	*k*_1_ (min^−1^)	0.0052	0.0050
	*R* ^2^	0.9679	0.9425
Pseudo-second-order	*q_e_* (mg·g^−1^)	61.27	106.31
	*k*_2_ (g·mg^−1^·min^−1^)	0.0001	6.61 × 10^−5^
	*R* ^2^	0.9872	0.9772

**Table 3:** Cu(II) and Pb(II) adsorption capacities of various adsorbents in single-component metal solutions.

Adsorbent	pH	Concentration (mg L^−1^)	Temperature (°C)	q_max_ (mg g^−1^)	References
Cu					
Activated carbon prepared from grape bagasse	5.0	10 to 100	25	37.17	[31]
γ-alumina nanoparticles	5.0	25 to 200	25	51.30	[32]
Natural manganese dioxide	5.52–5.10	158.87 (2.5 mmol L^−1^)	23	54.35	[27]
Groundwater treatment sludge-derived manganese dioxide	5.0	10 to 200	25	119.90	This study
Pb					
Biochar and activated carbon from cigarettes wastes	5.0	5 to 300	25	23.70 (biochar) 71.43 (activated carbon)	[33]
γ-Alumina	5.0	10 to 100	25	65.67	[34]
Manganese oxides recovered from spent alkaline and Zn/C batteries		5 to 100	25	6.04	[35]
Groundwater treatment sludge-derived manganese dioxide	5.0	10 to 200	25	177.89	This study

**Table 4:** Thermodynamic parameters for metal uptake.

Adsorbate	Temperature (°C)	Δ*G°* (kJ mol^−1^)	Δ*H°* (kJ mol^−1^)	Δ*S°* (kJ mol^−1^ K^−1^)	*E_a_* (kJ mol^−1^)
Cu(II)	25	−18.030	65.29	0.2798	146.76
	30	−19.420			
	35	−20.146			
Pb(II)	25	−22.588	37.62	0.2019	130.13
	30	−23.175			
	35	−23.809			

## Data Availability

Data is contained within the article.
